# Timely surgical intervention and risk stratification in patients with Ebstein anomaly: a 20-year retrospective cohort study

**DOI:** 10.1097/JS9.0000000000004760

**Published:** 2026-01-27

**Authors:** Yu Zhu, Zhao Jian, Ruiyan Ma, Yingbin Xiao

**Affiliations:** a Department of Cardiovascular Surgery, The Second Affiliated Hospital, Army Medical University, Chongqing, China; b Department of Cardiovascular Surgery, Hainan Hospital of Chinese PLA General Hospital, Sanya, Hainan Province, China

**Keywords:** cohort studies, Ebstein anomaly, nomograms, risk assessment, surgical intervention, treatment outcome

## Abstract

**Background:**

The optimal timing and choice of surgery for Ebstein anomaly (EA), a complex congenital heart defect, remain challenging due to the heterogeneity and lack of robust long-term data on EA. In this study, we aimed to evaluate the long-term outcomes of surgical management in patients with EA, identify the prognostic risk factors, and develop a predictive model.

**Materials and Methods:**

We conducted a retrospective analysis of data from 332 patients with EA who were treated at a tertiary center between January 2000 and December 2021. Among them, 269 underwent surgery: tricuspid valve repairs, 150; replacements, 77; and isolated bidirectional Glenn procedures, 42. Additionally, 70 patients received a concomitant Glenn shunt during valve surgery, resulting in a total of 112 Glenn procedures. The median follow-up was 10.12 years. The primary outcomes were freedom from reoperation and medical interventions. A predictive nomogram was developed using least absolute shrinkage and selection operator regression and internally validated.

**Results:**

The early surgical mortality rate was 2.60%. Postoperative complications occurred in 15.24% of the patients, with renal failure (4.83%) and arrhythmias (2.23%) being the most common. During a median follow-up of 10.12 years, the freedom from operation rates were 97.95, 92.48, 87.04, and 83.22% at 5, 10, 15, and 20 years, respectively. However, freedom from medical intervention showed a progressive decline (94.34% at 5 years vs. 62.31% at 20). Multivariable Cox regression analysis identified preoperative hepatic congestion [hazard ratio (HR) = 3.042], Wolff–Parkinson–White (WPW) syndrome (HR = 3.463), and elevated alanine aminotransferase (ALT) level (HR = 1.023) as independent risk factors for surgery. The concomitant bidirectional Glenn procedure was associated with a significantly reduced risk of both reoperation (HR = 0.160) and medical intervention (HR = 0.259). Patients requiring interventions showed significantly worse physical and emotional quality-of-life scores than did those who were event-free (*P* < 0.05).

**Conclusion:**

Timely surgical intervention guided by preoperative risk stratification optimizes the long-term outcomes of EA. The proposed nomogram was a practical tool for individualized risk assessment, supporting clinical decision making in patients with this complex condition.

## Introduction

Ebstein anomaly (EA) is a congenital malformation characterized by tricuspid valve insufficiency and right ventricular (RV) myopathy. It encompasses a spectrum of anatomic variants associated with differing pathophysiologies and prognoses^[1]^. Disease severity depends on the degree of septal leaflet displacement and morphological tricuspid valve dysplasia, leading to right atrioventricular junction expansion and RV failure^[2]^. Observations and medical management are typically recommended for mildly affected or asymptomatic patients. Nonetheless, surgical intervention should be considered for managing patients experiencing progressive cyanosis, decreased exercise tolerance, or arrhythmia^[3]^. Operative treatment has been shown to substantially improve long-term survival in these patients, including those with moderate or severe leaflet displacement^[4]^.

**HIGHLIGHTS**

- Reports long-term outcomes from a large surgical cohort (*n* = 332) of Ebstein anomaly with >10-year median follow-up.
- Identifies independent risk factors for reoperation: preoperative hepatic congestion, WPW syndrome, and elevated ALT.
- Demonstrates the concomitant bidirectional Glenn procedure as a significant protective factor, markedly reducing reoperation risk.
- Supports proactive intervention upon signs of right heart failure or hepatic congestion to optimize outcomes.
- Provides a practical nomogram for preoperative risk stratification and evidence-based surgical planning.

Surgical correction typically comprises tricuspid valve repair or replacement, RV plication, right atrial reduction, and atrial septal defect closure or subtotal closure^[5]^. Tricuspid valve repair is now considered the goal of surgical therapy when feasible. Alternatively, tricuspid valve replacement (TVR) is a safe and effective option in patients with severe leaflet displacement^[2]^. Furthermore, concomitant antiarrhythmic procedures should be considered during surgery, given their favorable long-term effects^[6]^. Specifically, the “One and a half ventricle repair” is selectively performed for cardiac decompensation owing to impaired right heart function^[7]^. However, no unified guidelines exist for surgical strategies given the variable pathoanatomical patterns of EA. Inadequate understanding of malformations and suboptimal surgical techniques could lead to postoperative morbidity and poor long-term outcomes.

Nevertheless, the long-term outcomes of tricuspid valve surgery in patients with EA remain a key focus. Addressing tricuspid insufficiency postoperatively remains challenging^[8]^. Data on postoperative outcomes in patients with EA, particularly those with severe tricuspid valve deformity, remain limited. Therefore, in this study, we aimed to investigate the long-term outcomes in a large EA cohort, identify predictors of adverse events, and develop a clinical risk prediction model to guide the timing and selection of surgical intervention.

There has been little information on reporting outcomes in patients with EA following surgery, particularly those with severe deformity of the tricuspid valve. However, the existing large-scale studies reporting outcomes are predominantly derived from Western national registries^[9–11]^. Crucially, the generalizability of these findings in Western studies to Asian populations is uncertain, as significant disparities are anticipated in key areas such as: first, an older age at presentation, influenced by distinct healthcare access pathways. Second, a differing disease spectrum, resulted from skewing toward advanced-stage adults due to the widespread adoption of prenatal screening for reducing severe neonatal cases. Third, consequently unique profile of risk factors could influence long-term outcomes. Therefore, in this study, we aimed to develop a clinically applicable risk-prediction model derived from and tailored to this specific patient population.

This cohort study was conducted in accordance with the STROCSS guidelines^[12]^.

## Methods

### Ethics

This single-center retrospective cohort study was conducted in accordance with the ethical principles of the Declaration of Helsinki. The study protocol was reviewed and approved by the Medical Ethics Committee of the Second Affiliated Hospital, Army Medical University (Approval No. 2024-Yan-061-01), which waived the requirement for informed consent due to retrospective analysis of anonymized clinical data. Verbal informed consent was obtained from the participants for prospective quality-of-life (QoL) assessments conducted during follow-up.

### Patients

We enrolled patients with a confirmed EA diagnosis between January 2000 and December 2021 in a retrospective follow-up study. The diagnosis of EA was confirmed preoperatively by echocardiography. Other baseline investigations, including electrocardiography, chest radiography, and 24-h Holter monitoring, were used as ancillary diagnostic tools. Patients with pulmonary atresia and an intact ventricular septum or other major associated cardiac anomalies were excluded. All cases were retrospectively reviewed using a standardized data collection protocol. The following data were collected: (1) baseline patient characteristics and preoperative clinical profiles; (2) intraoperative data; (3) postoperative data and operative mortality; (4) long-term follow-up data; and (5) late operations and interventions.

The unoperated control group comprised all patients with a confirmed EA diagnosis who did not undergo cardiac surgery. This group was inherently heterogeneous, including patients with mild anatomy and minimal symptoms, wherein surgery was not indicated and those with severe end-stage disease (e.g., fixed pulmonary hypertension, severe RV dysfunction) who were deemed inoperable due to excessive risk. This composition reflects the real-world clinical decision-making spectrum of EA.

### Diagnostic criteria

All patients were preoperatively diagnosed using a standardized multimodal protocol. The primary tool was comprehensive transthoracic echocardiography, with the key diagnostic criterion being an apical displacement of the septal tricuspid leaflet ≥8 mm/m^2^^[13]^. Additional echocardiographic assessments were used for quantifying the degree of RV atrialization, tricuspid regurgitation (TR) severity, and RV function^[14]^. Echocardiography was systematically supplemented by electrocardiogram (ECG), chest radiography, and 24-h Holter monitoring for assessing arrhythmias and pre-excitation, thereby fully characterizing the clinical profile^[15]^.

Cardiovascular magnetic resonance and cardiac catheterization are recognized for their complementary roles in quantifying RV size/function and assessing pulmonary hypertension, respectively^[16,17]^. However, owing to regional healthcare resource constraints during the study period, these advanced modalities were not routinely performed in all patients. The primary diagnostic foundation, comprehensive echocardiography, ECG, and Holter monitoring were rigorously applied to all included patients to ensure a consistent baseline diagnosis.

### Indication for surgery and surgical techniques

The natural history of the EA is influenced by the degree of tricuspid valve deformity. A 20-year follow-up study of unoperated patients with EA reported less than 40% survival in those with moderate or severe leaflet displacement^[18]^. However, surgical treatment significantly improved long-term outcomes. Surgical intervention was indicated for managing patients with severe TR, cyanosis, dyspnea, symptoms of right heart failure, progressive cardiac dilation, or associated cardiac defects^[3]^. Patients were stratified into surgical subgroups based on intraoperative findings: tricuspid valve plasty (TVP) (*n* = 150) was performed when anatomical repair was feasible; TVR (*n* = 77) was reserved for severe leaflet dysplasia or failed repair; and a bidirectional Glenn procedure (*n* = 112) was used for significant RV dysfunction or failure to wean from cardiopulmonary bypass.

The surgical techniques have been previously described in detail^[19]^. All operations were performed via the median sternotomy approach. Cardiopulmonary bypass was established with aortic and bicaval cannulation, following heparinization. Myocardial protection was achieved by aortic cross-clamping and infusion of cold blood cardioplegia. The specific procedures performed included: atrial septal defect/patent foramen ovale closure, correction of associated defects (e.g., ventricular septal defect or pulmonary stenosis), plication of the atrialized right ventricle, and tricuspid valve repair or replacement.

### Follow-up clinical assessment

Postoperative patients were regularly followed up through clinic visits. ECG or Holter monitoring was performed when arrhythmia recurrence was suspected. Patients underwent periodic echocardiography to assess tricuspid valve function. TR was graded from 1 to 4 using established methods^[18,19]^. The Minnesota Living with Heart Failure Questionnaire (MLHFQ) was completed by the patients or their caregivers during follow-up visits^[20]^. Functional status was assessed using the New York Heart Association (NYHA) classification system. Causes of death and indications for reintervention were ascertained from hospital records.

### Definition of primary outcomes

Regarding the primary outcomes: freedom from operation was defined as the time from the index surgery to the first subsequent cardiac surgical procedure involving the tricuspid valve, right ventricle, or any concomitant procedure performed during the initial surgery. Percutaneous interventions were not classified as reoperation. Freedom from medical intervention was a composite endpoint defined as the time from the index surgery to the first occurrence of any of the following events: cardiac reoperation (as defined above), initiation of or a sustained escalation in diuretic therapy specifically for right heart failure symptoms, or the need for new antiarrhythmic therapy or an ablation procedure for a clinically significant arrhythmia.

### Statistics

Analyses were performed using SPSS Statistics (version 26.0; IBM Corp., Armonk, New York, USA) and Prism 8.0 software (GraphPad Software, San Diego, California, USA). The proportion of missing data for each key variable is presented in Supplemental Digital Content Table S1, available at: http://links.lww.com/JS9/G711. Given the low overall proportion of missing data (<5% for all continuous variables) and following the recommended practices to reduce bias and preserve statistical power, multiple imputation was employed for continuous variables^[21]^. Predictive mean matching in SPSS was used to generate five imputed datasets. The imputation model included all variables included in the subsequent primary analysis. None of the categorical variables in the baseline or operative datasets required imputation, because they had complete data. Categorical variables from follow-up assessments with missing data were handled using complete-case analysis for their respective analyses^[22]^. The Kolmogorov–Smirnov test was used for evaluating the normal distribution of continuous variables. Continuous data are expressed both as the mean ± standard deviation and median (interquartile range, Q1, Q3). They were analyzed with one-way analysis of variance followed by the Bonferroni test when making multiple comparisons, while the Wilcoxon rank-sum test was used for analysis of continuous data with a non-normal distribution. Categorical variables are expressed as numbers and percentages and were analyzed using the chi-square test or Fisher’s exact test. Risk factors for early and late mortality or reinterventions were identified by univariate and multivariate analyses and analyzed using Cox regression. Survival and freedom from reoperation were estimated using the Kaplan–Meier method; intergroup comparisons were performed using the log-rank test. All statistical tests were two-sided; only differences with a *P* value of less than 0.05 were considered statistically significant.

Independent variables included age at surgery, patient sex, body weight, body height, body mass index (BMI), NYHA classification of heart failure, preoperative cyanosis or palpitate, preoperative liver function assessment, cardiothoracic ratio (CTR) on chest radiographic analysis, presence and type of preoperative arrhythmia, rhythm on electrocardiographic analysis, Doppler echocardiographic assessment of cardiac architecture and valve function, surgical approach, follow-up years, and post-surgery status of the patients.

To facilitate clinical application, a predictive nomogram for freedom from intervention and freedom from operation was developed and internally validated by the least absolute shrinkage and selection operator regression and bootstrap resampling. The prognostic performance of the nomogram was rigorously evaluated through discrimination analysis and calibration validations. Discrimination capacity was quantified using the area under the receiver operating characteristic curve with the following interpretation guidelines: 0.5 = random chance and 1 = perfect discrimination. Calibration accuracy was assessed by visual examination of calibration plots with 45-degree reference lines. Quantitative comparison between the predicted probabilities and observed frequencies was performed. Internal validation employed 1000 bootstrap resamples to estimate the model’s optimism-corrected predictive accuracy. Decision curve analysis was subsequently conducted to evaluate clinical utility across the probability thresholds. Regarding clinical implementation, an interactive web-based nomogram was developed using the Shiny package (v0.13.2.26), hosted on the RStudio Server. All statistical computations were performed in the R statistical environment (version 4.5.0; 2025-04-11) with the following critical packages: rms for regression modeling, DynNom for dynamic visualization, and rmda for decision curve analysis.

## Results

### Study population and patient characteristics

Substantial baseline differences were found between the surgical and non-surgical groups, as anticipated in the non-randomized cohort (Supplemental Digital Content Table S2, available at: http://links.lww.com/JS9/G711 and Supplemental Digital Content Table S3, available at: http://links.lww.com/JS9/G711). Consistent with the clinical rationale, the patients selected for surgery were generally younger and had more severe TR but better-preserved end-organ function, representing a population wherein a corrective procedure was deemed feasible and beneficial. However, the nonsurgical group included a mix of very mild and high-risk patients. These differences validated the presence of a significant selection bias, which was the primary confounding factor adjusted in our subsequent multivariable analyses.

**Figure 1.**
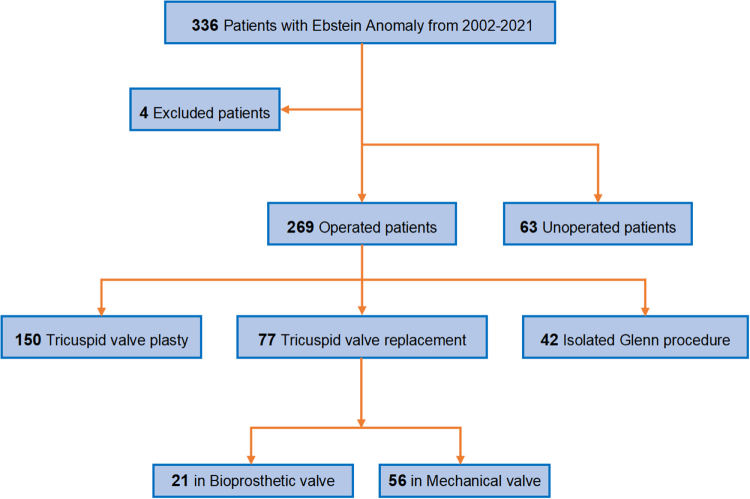
Flow diagram of participant selection.

This retrospective study included 336 EA patients. Four patients were excluded due to missing data. Among the remaining 332 patients, most underwent RV plication, right atrial reduction, or atrial septal closure. Of these, 150 underwent tricuspid valve repair. Seventy-seven patients underwent TVR. A bidirectional Glenn procedure was performed in 112 patients with planned or unplanned right heart failure. Sixty-three patients did not undergo cardiac surgery, because they did not meet the indications (Fig. 1).

The mean age of all participants was 29.60 ± 17.24 years (range, 6 months–66 years); 227 (68.37%) were female patients. Preoperative symptoms were common: 56 (16.87%) patients had cyanosis, and 141 (42.47%) had palpitations or syncope. All patients had reduced functional status preoperatively: 140 (42.17%) were NYHA class III and 27 (8.13%) were class IV. Chest radiography, ECG, and echocardiography were mandatory tests that must be conducted upon hospital admission. The CTR was 0.62 ± 0.10 in all patients according to chest radiographs. The ECG showed that 33 patients (9.94%) experienced atrial fibrillation or atrial flutter, while 24 patients (7.23%) had Wolff–Parkinson–White (WPW) syndrome. Additionally, on ECG, 119 patients (35.84%) exhibited incomplete or complete right bundle branch block, and 86 patients (25.90%) had right axis deviation (Supplemental Digital Content Table S2, available at: http://links.lww.com/JS9/G711). All patients were diagnosed by echocardiography. Severe tricuspid valve insufficiency occurred in 226 (68.07%) patients. In total, 168 patients had atrial septal defects. The concrete data are listed in Supplemental Digital Content Table S3, available at: http://links.lww.com/JS9/G711.

### Operative data

A total of 269 patients underwent surgery. Seventy-seven (28.62%) patients underwent valve replacement instead of failed repair; in parallel, 42 patients (15.61%) underwent isolated bidirectional cavopulmonary shunt (BCPS). Among the 77 valve replacements, biological prostheses were used in 21 cases (27.27%) (Fig. 1). The mean cardiopulmonary bypass and aortic cross-clamp times were 109.07 ± 37.29 and 68.74 ± 26.77 min, respectively (Supplemental Digital Content Table S4, available at: http://links.lww.com/JS9/G711).

### Operative mortality and morbidity

Operative mortality was 2.60% (7/269), occurring in three (2.00%), two (2.60%), and two (4.76%) patients who underwent repair, replacement, and isolated Glenn procedure, respectively. The primary causes of death were refractory low cardiac output syndrome and multiorgan failure. Major perioperative complications occurred in 41 patients (15.24%), including renal failure (*n* = 13, 4.83%), respiratory insufficiency (*n* = 3, 1.12%), and arrhythmia (*n* = 6, 2.23%). The blood product transfusion requirements and postoperative hospital stay were significantly longer in the valve replacement group (Supplemental Digital Content Table S5, available at: http://links.lww.com/JS9/G711).

All postoperative complications were comprehensively classified according to the Clavien–Dindo system^[23]^. Overall, 63 patients (23.42%) had no complications. This complete classification encompassed all grades of complications (Supplemental Digital Content Table S6, available at: http://links.lww.com/JS9/G711). We further investigated inter-complication relationships using a multivariable logistic regression model. Notably, no significant independent associations were identified, indicating that complications in this cohort did not follow a strong cascading pattern.

Multivariate analysis of univariate predictors showed that the CTR was a significant risk factor for early mortality. Regarding clinical interpretability, CTR was standardized (per 0.1 unit increase) using a sensitivity analysis, yielding an odds ratio (OR) of 3.21 [95% confidence interval (CI): 1.89–5.45; *P* < 0.001]. Right axis deviation on ECG was also a risk factor (OR = 6.12; 95% CI: 1.01–37.02; *P* = 0.049).

We also analyzed the association between postoperative biomarker levels and the occurrence of major in-hospital complications. No statistically significant differences were observed in Cardiac Troponin I (cTnI), creatine kinase-MB (CK-MB), and myoglobin levels between patients who experienced major complications and those who did not, whereas biomarker levels were elevated postoperatively (Supplemental Digital Content Table S7, available at: http://links.lww.com/JS9/G711).

### Long-term outcomes

Complete clinical follow-up data of 303 of 325 operative survivors (93.23%) were available. Baseline characteristics were comparable between patients with complete follow-up and those lost to follow-up (*n* = 22). No significant differences were observed (*P* > 0.05), suggesting that the loss to follow-up was random and unlikely to introduce a substantial bias. The average time was 11.01 ± 5.57 years (median: 10.12 years; range: 0.31–23.48 years).

During follow-up, four patients (5.19%) in the valve replacement group experienced anticoagulation-related bleeding or thromboembolic complications.

Postoperative TR showed marked improvement over the preoperative status at the final follow-up in most patients with EA. However, 85 patients had moderate or severe tricuspid valve insufficiency. RV function at the final follow-up was documented in 264 patients and graded as normal or mildly impaired in 179 (67.80%) (169 received surgical treatment, while 10 did not), moderately impaired in 80 patients and severely impaired in 5. Left ventricular function at the final follow-up was documented and graded as moderately or severely impaired in eight patients (seven received surgical treatment, while only one did not). Two patients exhibited biventricular dysfunction.

**Figure 2.**
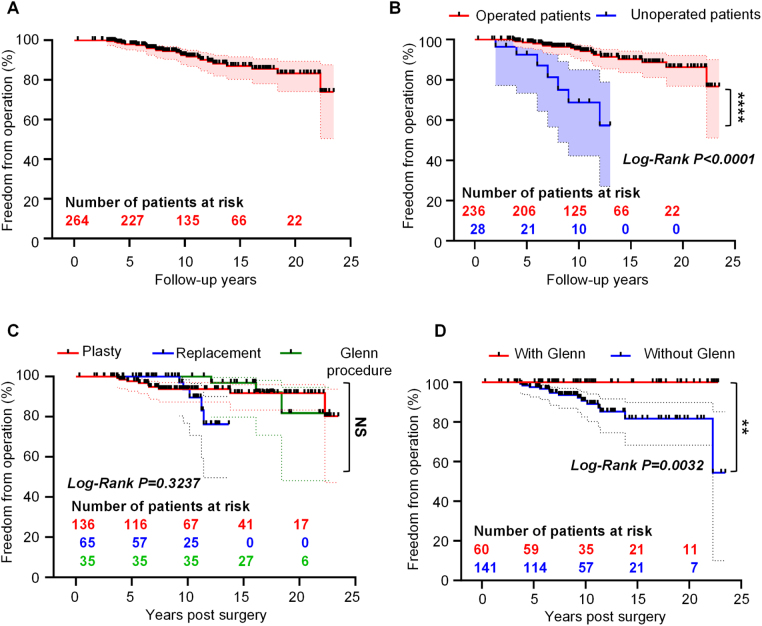
Kaplan–Meier curves of freedom from operation according to different groups. (A) Freedom from operation in patients with EA. (B) Freedom from operation in patients with EA after surgery compared with unoperated patients. (C) Long-term freedom from operation in the subgroups among operated patients with EA. (D) Freedom from operation in operated patients with EA with the Glenn procedure compared to those without the procedure. EA, Ebstein anomaly.

Regarding surgeries, in the whole cohort, the freedom from operation rate was 97.95% (95% CI: 95.14–99.14%) at 5 years, 92.48% (95% CI: 87.74–95.44%) at 10, 87.04% (95% CI: 80.27–91.60%) at 15, and 83.22% (95% CI: 74.09–89.36%) at 20 (Fig. 2A). Although late surgeries were required in 23 patients, only 13 of these patients underwent reoperation. Surgical patients demonstrated a reduced long-term risk of valve-related operation compared with their non-surgical counterparts [hazard ratio (HR) = 0.01801, 95% CI: 0.003377–0.09602] (Fig. 2B). Although no significant difference in long-term freedom from reoperation was observed among the three surgical strategies (log-rank, *P* = 0.3237) (Fig. 2C), their complication profiles substantially differed. Compared with the replacement group, patients in the repair group avoided the need for lifelong anticoagulation, resulting in a significantly lower incidence of anticoagulation-related events (0 vs. 5.2%, *P* < 0.01). Concomitant Glenn procedure was a significant independent protective factor against reoperation (HR = 0.19, 95% CI: 0.064–0.577) (Fig. 2D). This imprecision possibly reflects the selective application of this technique to a higher-risk profile, including 10 patients who required unplanned Glenn procedures for failure to wean from cardiopulmonary bypass. This selection bias is further suggested by a non-significant trend towards higher early mortality in the Glenn group (4.8 vs. 2.0% in the repair group).

**Figure 3.**
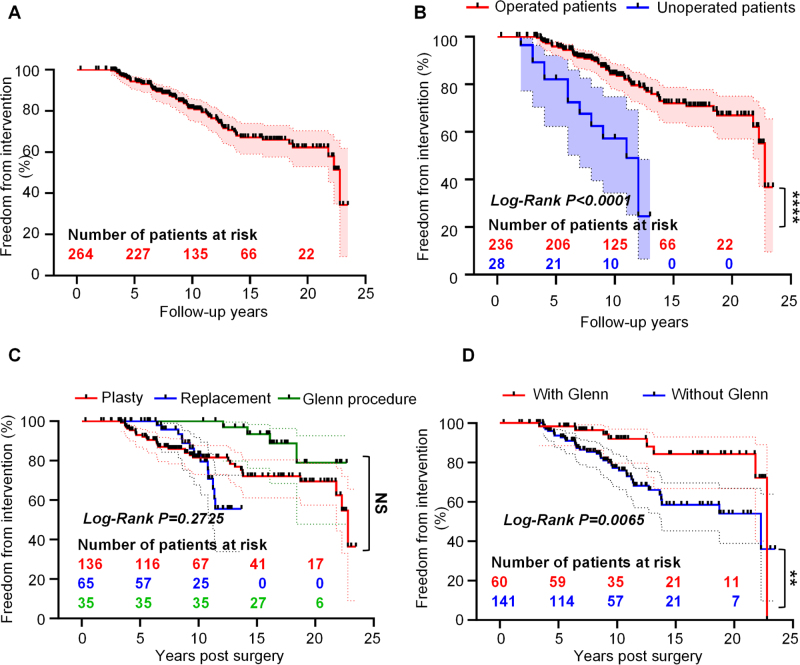
Kaplan–Meier curves of freedom from medical intervention according to different groups. (A) Freedom from medical intervention in patients with EA. (B) Freedom from medical intervention in patients with EA after surgery compared with unoperated patients. (C) Long-term freedom from medical intervention in subgroups among operated patients with EA. (D) Freedom from medical intervention for operated patients with EA with the Glenn procedure compared with those without the procedure. EA, Ebstein anomaly.

Considering intervention, the freedom of intervention rate was 94.34% (95% CI: 90.63–96.61%) at 5 years, 81.32% (95% CI: 75.31–86.00%) at 10, 67.08% (95% CI: 59.00–73.93%) at 15 and 62.31% (95% CI: 52.93–70.35%) at 20 (Fig. 3A). Medical treatment was required in 85 patients, including 23 patients who required reoperation, 58 patients who received diuretic therapy, and an additional 4 patients who required antiarrhythmic intervention. Surgical patients demonstrated a reduced long-term risk of medical intervention compared with their non-surgical counterparts (HR = 0.1610, 95% CI: 0.03070–0.8441) (Fig. 3B). No significant difference in long-term medical intervention rates was observed among the three surgical approaches in the operative cohort (log-rank, *P* = 0.2725) (Fig. 3C). However, concomitant Glenn procedure during the index operation significantly reduced the risk of late medical intervention (HR = 0.4238, 95% CI: 0.2285–0.7858) (Fig. 3D).

Given the observed baseline differences among the surgical groups, we performed multivariable Cox proportional hazards analyses to provide an adjusted comparison of the three primary surgical strategies (TVP, TVR, and isolated Glenn procedure). After adjusting for key confounders, including age, CTR, and preoperative TR severity, we found no statistically significant independent association between the choice of primary surgical strategy and risk of reoperation (TVR vs. TVP: HR = 0.85, 95% CI: 0.06–11.54, *P* = 0.900; isolated Glenn vs. TVP: HR = 1.01, 95% CI: 0.07–15.57, *P* = 0.994). The wide CIs reflect substantial statistical uncertainty in these estimates, possibly due to the limited number of events for this comparison. A similar lack of significant association was found regarding the outcome of freedom from medical interventions (Supplemental Digital Content Table S8, available at: http://links.lww.com/JS9/G711).

**Figure 4.**
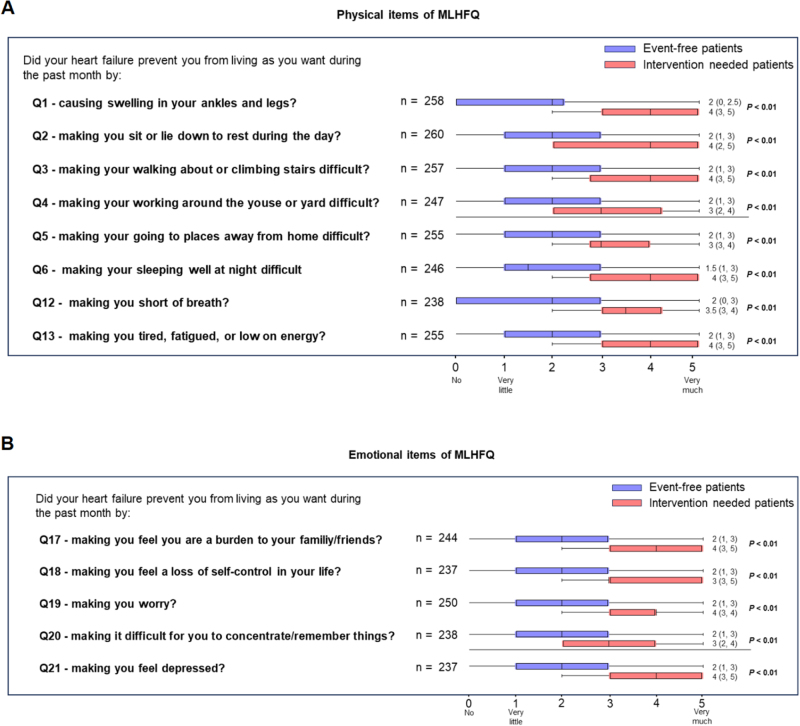
Change in the dimension of quality of life (QoL) in intervention needed patients and event-free patients. (A) Change in the physical dimension of QoL in the two groups. (B) Change in the emotional dimension in QoL in the two groups.

QoL was estimated by the MLHFQ score during follow-up. The physical subdomain of the MLHFQ consisted of eight items (Fig. 4A). The patients were stratified into two groups based on their current receipt of medical interventions. Unadjusted analysis revealed that patients requiring medical intervention had significantly worse scores in the physical domain of the MLHFQ compared with event-free survivors (Fig. 4A). This difference persisted using a multivariate linear regression model that adjusted for key baseline characteristics, including age and preoperative NYHA functional class, confirming that the need for intervention was independently associated with poorer physical QoL. The emotional subdomain of the MLHFQ consisted of five different items (Fig. 4B). Specifically, the five emotion-related items were impaired in patients requiring intervention rather than those who were free of events (Fig. 4B).

The risk factors for surgery in all cohorts were preoperative WPW syndrome (HR = 3.463, 95% CI: 1.344–8.91, *P* = 0.010), preoperative alanine aminotransferase (ALT) (HR = 1.023, 95% CI: 1.003–1.043, *P* = 0.021), preoperative liver congestion (HR = 3.042, 95% CI: 1.130–8.189, *P* = 0.028), surgical intervention (HR = 0.222, 95% CI: 0.082–0.602, *P* = 0.003), and bidirectional Glenn procedure (HR = 0.160, 95% CI: 0.044–0.584, *P* = 0.006) (Supplemental Digital Content Table S9, available at: http://links.lww.com/JS9/G711).

**Figure 5.**
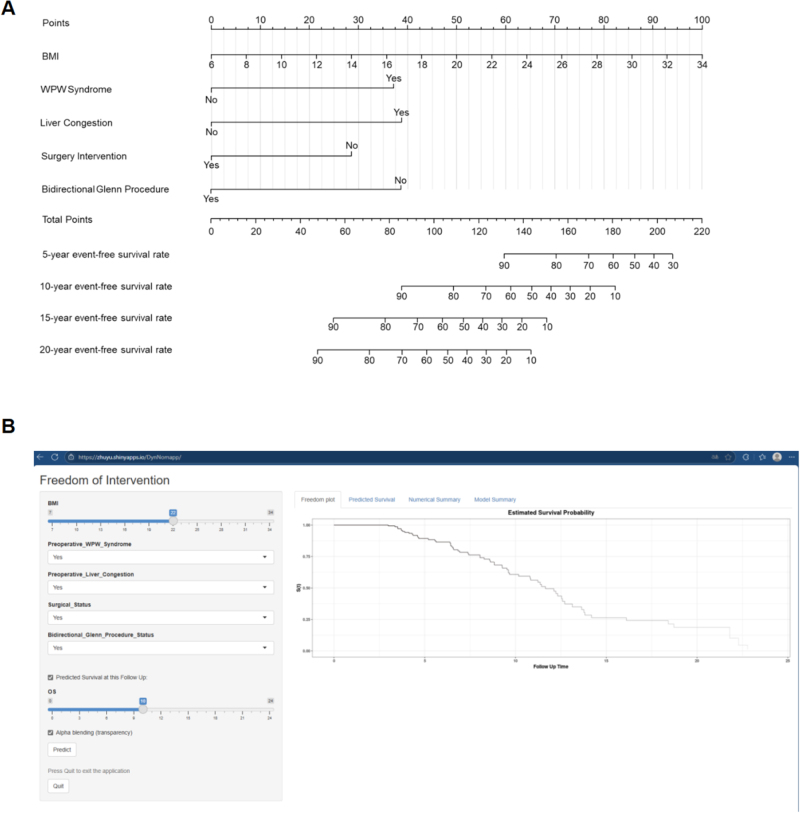
Established nomogram for the prediction of freedom from medical intervention in the follow-up years. (A) Static nomogram. (B) Online dynamic nomogram accessible at https://zhuyu.shinyapps.io/DynNomapp/, depicting an example for predicting the probability of freedom from medical intervention in the follow-up years in a patient with EA (BMI = 22.0, preoperative WPW syndrome and preoperative liver congestion, underwent surgical treatment and Glenn procedure). BMI, body mass index; EA, Ebstein anomaly; WPW, Wolff–Parkinson–White

Risk factors for intervention in all cohorts were BMI (HR = 1.112, 95% CI: 1.048–1.180, *P* < 0.001), preoperative WPW syndrome (HR = 3.402, 95% CI: 1.870–6.189, *P* < 0.001), preoperative liver congestion (HR = 3.277, 95% CI: 1.963–5.471, *P* < 0.001), surgical intervention (HR = 0.404, 95% CI: 0.215–0.758, *P* = 0.005), and bidirectional Glenn procedure (HR = 0.259, 95% CI: 0.131–0.515, *P* < 0.001) (Supplemental Digital Content Table S9, available at: http://links.lww.com/JS9/G711). The final Cox model, incorporating five independent predictors (BMI, preoperative WPW syndrome, preoperative liver congestion, surgical intervention, and bidirectional Glenn procedure), was developed as a simple-to-use nomogram (Fig. 5A). The available online (https://zhuyu.shinyapps.io/DynNomapp/) is screenshotted (Fig. 5B).

Risk factors for operation in operated patients were preoperative ALT (HR = 1.020, 95% CI: 1.000–1.041, *P* = 0.050), preoperative liver congestion (HR = 4.483, 95% CI: 1.460–13.761, *P* = 0.009), and bidirectional Glenn procedure (HR = 0.174, 95% CI: 0.047–0.644, *P* = 0.009).

The risk factors for intervention in patients undergoing surgery were preoperative WPW syndrome (HR = 2.101, 95% CI: 1.006–4.390, *P* = 0.048), preoperative liver congestion (HR = 3.386, 95% CI: 1.885–6.083, *P* < 0.001), preoperative left ventricular diameter (HR = 1.055, 95% CI: 1.007–1.104, *P* = 0.023), and bidirectional Glenn procedure (HR = 0.260, 95% CI: 0.131–0.517, *P* < 0.001).

Using sensitivity analysis on temporal bias, we investigated the potential temporal bias across the 20-year study period. Stratified by the median surgical year, the long-term outcomes were significantly superior in the early era (2000–2011) compared with the modern era (2012–2021) (Supplemental Digital Content Figure S1A, available at: http://links.lww.com/JS9/G711 and Supplemental Digital Content Figure S1B, available at: http://links.lww.com/JS9/G711). To elucidate this counterintuitive finding, we compared the baseline characteristics between eras (Supplemental Digital Content Table S10, available at: http://links.lww.com/JS9/G711). This analysis revealed that the modern era cohort represented a significantly higher-risk population, as evidenced by an older age (32.52 ± 16.58 vs. 23.14 ± 16.27 years, *P* < 0.001), higher prevalence of severe TR (80.38 vs. 64.86%, *P* = 0.004), elevated secondary pulmonary arterial pressure (37.70 ± 11.91 vs. 32.79 ± 9.50 mmHg, *P* = 0.002), and greater need for TVR (38.61% vs. 14.41%, *P* < 0.001). The concomitant bidirectional Glenn procedure was performed less frequently in the modern era (30.38 vs. 57.66%, *P* < 0.001), possibly reflecting its declining role as a planned strategy in an older, morphologically less suitable cohort. After adjusting for this era effect in the multivariable Cox model, the surgical era remained an independent risk factor; moreover, all previously identified prognostic factors retained their significance.

## Discussion

This single-center cohort study with long-term follow-up provided robust evidence that surgery significantly improved outcomes in patients with EA. Our findings are consistent with the recent 2025 American Association for Thoracic Surgery (AATS) Expert Consensus Document on EA management^[24]^.

Our data from an Asian tertiary center revealed important demographic differences from those of Western cohorts^[9–11]^. Widespread prenatal screening in China has reduced the incidence of severe neonatal EA, shifting our surgical population towards adults (mean age: 29.6 years), largely pre-screening era survivors or those with progressive, adult-onset disease. This explains the pronounced prognostic role of preoperative hepatic congestion in our model, reflecting the long-standing hemodynamic burden in adults. It also clarified an apparent contradiction: Although Swedish data show better survival in unoperated patients (possibly representing mild cases managed conservatively)^[9–11]^, our study demonstrated a clear protective effect of surgery in symptomatic adults, a population of whom robust surgical data are scarce. Thus, our work complements the pediatric-focused Western literature.

Our study also delineated key prognostic factors and provided a practical risk stratification tool. The timing and choice of surgery must be individualized based on the clinical status and valve morphology^[3,25]^. Available predictors have been summarized in previous studies to support the decision-making process in patients with EA in previous studies^[26]^. The tricuspid valve has historically been considered a “forgotten” valve. Recently, the pathophysiological and prognostic importance of the tricuspid valve and right ventricle has been increasingly recognized^[8,27]^. The use of isolated tricuspid valve surgery has increased, albeit with substantial morbidity and mortality^[28,29]^. Surgeons still face challenges in anatomically repairing the tricuspid valve and right ventricle, which is the most ideal procedure in patients with EA^[30,31]^. Surgery should not be excessively delayed in pediatric patients, whereas intervention timing in adults requires careful consideration^[24]^. Valve repair should be the preferred strategy when anatomically feasible, followed by replacement, to maximize improvement in TR and long-term prognosis^[10]^. Although the long-term freedom from reoperation was comparable across techniques, repair should be preferred when feasible, particularly in younger patients, as it avoids lifelong anticoagulation and its associated risks. Valve replacement is an effective option when repair is unachievable.

The compelling freedom from reoperation rates of 92.48% at 10 and 83.22% at 20 years extend beyond technical success. This durability provides a survival advantage, reduces healthcare burden, and crucially correlates with a better QoL. The superior MLHFQ scores in intervention-free patients empirically demonstrated that a stable clinical course correlated with better daily functioning and well-being. Thus, freedom from reoperation could serve as both a surgical benchmark and a patient-centered proxy for long-term health status.

Various techniques have focused on achieving near-anatomical and durable tricuspid valve repair^[4]^. Few comparative studies exist between different repair techniques (Danielson, Carpentier, Cone)^[25]^. Contemporary cone reconstruction has produced favorable results regarding mortality, valve function, and RV remodeling^[32,33]^. However, the potential contraindications have limited their application. Older age, secondary pulmonary hypertension, left ventricular failure, and severe annular dilation may impair the long-term repair outcomes^[17]^. Consistent with other studies, we found that preoperative ALT level, liver congestion, and the Glenn procedure were risk factors for reoperation. This confirms that preoperative functional status and TR severity are related to outcomes^[10]^. Additionally, 21 patients underwent tissue prostheses for valve replacement. This represents an alternative when repair is impossible; however, repair remains the first-line^[34,35]^.

Previous studies have shown that a BCPS provides RV volume offloading, reduces perioperative mortality, and improves tolerance of TR in high-risk patients with EA^[7,36]^. Concomitant BCPS is a useful adjunct in patients with EA with severe RV dilatation or dysfunction^[37]^. The procedure can be performed safely; however, it prolongs postoperative care and increases morbidity. Intermediate-term survival and QoL are good to excellent^[38]^. In our study, BCPS was used in 112 patients with severe right heart failure, including 10 unplanned cases of failure to wean from cardiopulmonary bypass. This strategy is more common in children than in adults due to long-term concerns about paradoxical embolism^[17]^. Our analysis confirmed that BCPS is a protective factor for long-term prognosis. The Glenn procedure requires careful interpretation. The isolated Glenn procedure showed no independent association with the outcomes, whereas the concomitant Glenn procedure was protective. This discrepancy reflects patient selection: the isolated procedure may be reserved for higher-risk patients not indicated for valve surgery, whereas the concomitant procedure provides strategic ventricular unloading in selected cases, realizing physiological benefits that alter the long-term course. This effect is explained by the following mechanism: effective RV volume unloading reduces preload and wall stress, potentially facilitating reverse remodeling and enhancing repair durability^[7]^, thereby interrupting the cycle of progressive right heart failure^[36]^.

In our cohort, the Glenn procedure for ventricular unloading offered long-term benefits in selected high-risk patients. However, it was associated with a higher risk of perioperative complications. Therefore, this technique should be reserved for severe RV dysfunction and not used as a routine adjunct. This approach aligns with the AATS Consensus, endorsing selective use for significant RV dysfunction to reduce preload^[24]^.

Our analysis of postoperative biomarkers provided novel insights into risk stratification in EA. Contrary to the established literature on left-sided valvular surgery^[39]^, the markers of direct myocardial injury (cTnI, CK-MB, myoglobin) were not predictive of major early complications in our cohort. This discrepancy might be attributed to the unique right-heart-dominant pathophysiology of EA. The chronic, adaptive nature of RV remodeling and dysfunction in this condition might fundamentally differ from the acute ischemic injury typically reflected by these biomarkers, which are more sensitive to left ventricular damage. Therefore, the magnitude of perioperative myocyte necrosis, as quantified by these conventional biomarkers, might not be the dominant driver of early adverse outcomes in this specific population. This finding underscores the need for evaluating the risk of EA from the perspective of its distinct RV physiology and highlights the potential limitation of applying risk models derived from left-heart disease.

A key innovation is the development and validation of an easy-to-use nomogram (available online) that integrates five predictors to estimate the individual risk of future interventions. This model facilitates a more precise surgical approach. Highlighting how this model differs from and improves upon the existing risk assessment approaches is crucial. Several established risk models are currently in use^[40]^. These range from general-purpose scores such as the Society of Thoracic Surgeons (STS) score to the newer procedure-specific TRI-SCORE, which predicts in-hospital mortality for isolated tricuspid valve surgery^[41–43]^. However, a validated tool for predicting long-term reintervention risk in patients with EA is lacking. Our nomogram fills this critical gap. It is the first model tailor-made for the long-term management of the EA, integrating disease-specific predictors (e.g., hepatic congestion and WPW syndrome) that are absent from generic models. Therefore, its primary utility and novelty lie in providing personalized, quantifiable long-term risk estimates, which can facilitate shared decision making between surgeons and patients, particularly in the timing of operation before the onset of irreversible complications, such as significant cardiomegaly.

Furthermore, the comprehensive classification of complications provided a nuanced view of postoperative morbidity in our EA cohort. Major complications, such as renal failure and arrhythmias, represent significant clinical events that invariably prolong intensive care unit stay and increase healthcare resource utilization^[44,45]^. At our institution, the management of these complications is protocol-driven and multidisciplinary: severe renal dysfunction triggers nephrology consultation with the liberal use of continuous renal replacement therapy for diuretic-refractory fluid overload, whereas significant arrhythmias prompt immediate electrophysiology consultation for optimized medical therapy or electrical cardioversion. Of note, contrary to the postoperative morbid spiral often described in other cardiac surgery populations, our rigorous subgroup analysis did not identify significant independent associations between the different major complications. This might reflect the unique right-heart-dominant pathophysiology of the EA, which potentially uncouples the classic cardiorenal and cardiopulmonary interactions observed in left heart failure. Furthermore, when assessed for their impact on the ultimate patient-centered outcome, major complications in our cohort were not independent predictors of worse long-term freedom from medical intervention. This crucial finding suggests that while representing a major immediate clinical challenge, these complications do not necessarily dictate the ultimate long-term fate in patients with EA, possibly owing to successful containment and management through aggressive, proactive institutional protocols.

The superior long-term outcomes observed in the early era are not an indicator of technical regression but rather a direct reflection of a fundamental shift in our patient population. The objective data demonstrated a deliberate expansion of our surgical indications to include older, higher-risk patients with more advanced disease, characterized by a higher burden of severe TR, secondary pulmonary hypertension, and valve morphology often deemed irreparable. Although refinements in surgical techniques and perioperative care have undoubtedly occurred, their beneficial effect on long-term durability appears to have been offset by the substantially increased intrinsic risk in the patient cohort in the modern era. This observation underscores the importance of risk-adjusted outcomes in long-term surgical studies. It also redefines the measure of surgical progress: from solely improving outcomes in classic, lower-risk anatomy to successfully extending the feasibility of surgery to a more complex and historically underserved patient population, even when their long-term trajectory remains challenged by their advanced disease state.

The interplay among postoperative morbidity, long-term clinical trajectory, and ultimate QoL is central to understanding the holistic impact of surgery. Although our analysis did not identify a strong “postoperative morbid spiral” of inter-complication relationships, the occurrence of a major complication represents a significant physiological insult that can diminish a patient’s functional reserve. This compromised reserve might translate into a more challenging long-term course, often evidenced by a persistent need for medical intervention such as diuretic therapy for subclinical heart failure. Our findings regarding QoL bring this chain of events to its patient-centered conclusion: the need for such reintervention has emerged as a powerful, independent predictor of poorer daily well-being. This was empirically demonstrated by its strong association with worse scores in both the physical and emotional domains of the MLHFQ. Therefore, the long-term requirement for medical care is not only a merely clinical endpoint, but also a potent marker of the disease’s intrusion into the patients’ daily life, effectively linking the early postoperative experience with the ultimate goal of restoring functional health and well-being.

Contemporary EA management emphasizes multidisciplinary collaboration for timing interventions and personalizing therapy^[11]^. Long-term outcomes are determined by clinical status and cardiac enlargement during initial surgery^[46]^. The pathophysiological progression of TR involves progressive multiorgan dysfunction with frank impairment of organ systems, signifying advanced-stage TR lesions^[47]^. These manifestations constitute “TR syndrome,” a framework for evaluating systemic sequelae and guiding management^[27]^. The NYHA classification has limited congruence with right heart failure pathophysiology in TR, given the distinct hemodynamics and RV–pulmonary uncoupling^[48]^. RV functional assessment remains challenging, necessitating the use of novel biomarkers to evaluate ventriculoarterial coupling and detect decompensation risk^[27]^.

Notably, cardiac MRI is considered a reference standard for evaluating RV size and volume, and can be used to quantify the severity of TR^[8]^. The STS-updated tricuspid valve surgical risk algorithm has been validated for preoperative risk stratification in EA populations, thereby enhancing clinical decision-making precision (https://isolatedtvsurgcalcm.research.sts.org/). In addition, transcatheter tricuspid interventions have emerged as an alternative treatment for TR^[49,50]^. Promising preliminary results have been achieved in several products, which might be a revolution in the reoperation of tricuspid valves^[51,52]^.

The long-term outcomes in this cohort reflected the interconnected stages of the patient journey, with QoL as the ultimate endpoint. Durable freedom from reoperation provides more than technical success; it confers a survival advantage and establishes a stable anatomical foundation for superior daily function. Conversely, major postoperative complications represent a physiological insult, heralding a more challenging course often requiring ongoing medical therapy, which is a strong predictor of diminished QoL. Thus, the entire management strategy for EA, from preoperative risk stratification to long-term surveillance, serves a singular, patient-centered goal: achieving a functional state that enables not only survival, but also improved QoL.

### Limitations

This study has several inherent limitations. First, as a retrospective analysis from a single tertiary referral center, our cohort was subject to selection bias, as evidenced by the baseline differences between the groups. We explicitly acknowledge that these differences might reflect the presence of selection bias. We have taken care to clearly define the control group and employed multivariable analysis to statistically adjust for these measured confounders. However, the possibility of residual confounding owing to unmeasured factors cannot be entirely excluded. Second, the 30-year study span introduces significant temporal bias; profound evolutions of surgical techniques, perioperative care, and patient selection criteria preclude the direct comparison of outcomes across different eras.

The sample size, which is substantial for rare diseases, imposed specific analytical constraints. The wide CIs in our adjusted comparisons of surgical strategies highlight the limited statistical power of these subgroup analyses, underscoring the challenge of achieving adequately powered comparisons in single-center studies of rare conditions.

Finally, the 93.23% follow-up rate, while being adequate, risks nonresponse bias, and retrospective echocardiographic assessments by different operators might have introduced heterogeneity.

## Conclusion

This large cohort study demonstrated that contemporary surgical management of EA was associated with low early mortality and provided durable long-term freedom from reoperation. Valve reconstruction was feasible in most patients. The outcomes showed acceptable freedom from reoperation and low reintervention rates. Risk was determined by the clinical status at the time of surgery. This study advocated for timely and individualized management. Selective use of the Glenn procedure could benefit high-risk patients; valve repair should be pursued when feasible. The proposed nomogram might offer a novel and practical tool for risk assessment and surgical timing, potentially improving long-term survival and QoL .

## Data Availability

All data generated or analyzed during this study are included in this article and its supplemental material. Furthermore, the underlying dataset supporting the findings of this study has been deposited in the China National Center for Bioinformation and is available at https://ngdc.cncb.ac.cn/gsub/. Meanwhile, they are also available from the corresponding author on reasonable request.

## Research registration unique identifying number (UIN)

This study was registered at the Chinese Clinical Trial Registry (ChiCTR) with Registration number (ChiCTR2500112980).
